# Endometrial assessment in abnormal uterine bleeding: transvaginal ultrasound alone may not be good enough

**DOI:** 10.1002/uog.70126

**Published:** 2025-11-04

**Authors:** S. Nijjar, S. Kastora, A. Bajrami, S. Solangon, M. Widschwendter, D. Jurkovic

**Affiliations:** ^1^ EGA Institute for Women's Health, Faculty of Population Health Sciences University College London London UK; ^2^ Department of Women's Cancer, EGA Institute for Women's Health, Faculty of Population Health Sciences University College London London UK; ^3^ European Translational Oncology Prevention and Screening Institute University of Innsbruck Tirol Austria; ^4^ Research Institute for Biomedical Aging Research University of Innsbruck Innsbruck Austria

**Keywords:** angle of insonation, endometrial thickness, multimodal sonography, transvaginal ultrasound, uterine bleeding

## Abstract

**Objective:**

To evaluate the proportion of optimal assessments of endometrial thickness (ET) achieved by transvaginal ultrasound (TVS) compared with a multimodal (MM) sonographic approach in women with abnormal uterine bleeding (AUB).

**Methods:**

This prospective observational study was a subset of the EPI‐SURE study conducted between June and November 2022 in a tertiary gynecology diagnostic referral center in the UK. All women aged ≥ 45 years with AUB who underwent TVS assessment of the endometrium were included. If the endometrium could not be visualized optimally on TVS, women were offered another method of endometrial assessment (MM approach), namely transabdominal ultrasound, transrectal ultrasound and/or saline contrast sonohysterography. We assessed the diagnostic performance of TVS compared with the MM approach in terms of the quality of endometrial visualization and measurement of ET, and used uni‐ and multivariable logistic regression analyses to evaluate associations between patient characteristics and imaging outcome.

**Results:**

We included 387 women who underwent TVS for endometrial assessment. In 245/387 (63.3%) cases, ET measurement was deemed optimal on TVS. The most common standalone reasons for suboptimal TVS were adenomyosis (37/142, 26.1%) and unfavorable uterine position (37/142, 26.1%), followed by uterine fibroids (21/142, 14.8%) and other pathologies or presence of intrauterine system (6/142, 4.2%). In 41/142 (28.9%) women, the reason for suboptimal TVS was multifactorial. The median angle of insonation on TVS in women with an unfavorable uterine position was 33° (interquartile range (IQR), 15–52°), which increased to 86° (IQR, 78–90°) on MM imaging (*P* < 0.0001). In cases with suboptimal TVS due to inadequate imaging of the uterine cavity and/or a suboptimal angle of insonation, ET measurement was significantly higher on TVS compared with the MM approach (median difference, −1.3 (95% CI, −1.7 to −0.9) mm; *P* < 0.0001). On multivariable analysis, factors associated significantly with suboptimal TVS included fibroids distorting the uterine cavity and younger age; women with fibroids had 10 times the odds of a suboptimal TVS assessment of the endometrium compared to those without. Cavity‐distorting fibroids and higher body mass index were associated significantly with suboptimal MM imaging on multivariable analysis, with fibroids increasing the odds of this outcome by 4.2 times. A prespecified ET cut‐off of ≥ 4.5 mm yielded a specificity of 85.8% (95% CI, 78.0–91.7%) with the MM approach for the diagnosis of endometrial cancer. Using the MM sonographic approach avoided the need for further procedures to assess the endometrium in 118/387 (30.5%) cases.

**Conclusion:**

The MM sonographic approach is a better way to assess the endometrium compared with TVS alone in women presenting with AUB. The MM approach improved endometrial assessment by optimizing the angle of insonation and visualization of the uterine cavity, which resulted in significantly lower ET measurements and a higher specificity for endometrial cancer, thereby potentially reducing the number of invasive interventions, such as hysteroscopy and endometrial biopsy. © 2025 The Author(s). *Ultrasound in Obstetrics & Gynecology* published by John Wiley & Sons Ltd on behalf of International Society of Ultrasound in Obstetrics and Gynecology.

## INTRODUCTION

Endometrial cancer is the most common gynecological cancer in the UK and its incidence continues to rise^1^. Timely diagnosis is important in improving prognosis. The British Gynaecological Cancer Society recommends transvaginal ultrasound (TVS) as the first‐line investigation for assessing endometrial thickness (ET) in women with abnormal uterine bleeding (AUB), including postmenopausal bleeding^2^. If the endometrium is thickened, more invasive investigations, including endometrial biopsy and/or hysteroscopy, are recommended. An ET cut‐off of ≥ 3–5 mm is used to classify women as high risk^2^, ^3^, ^4^, ^5^. Several studies have shown that the diagnostic accuracy of ultrasound in detecting the presence or absence of endometrial cancer can be improved by using subjective pattern recognition, which assesses endometrial morphological features and vascular pattern on Doppler^6^, ^7^. While these methods have been shown to improve the sensitivity of ultrasound for diagnosing endometrial cancer, the specificity remains relatively low and, as a result, many women undergo unnecessary further investigation. Previous studies have shown that TVS remains suboptimal in assessing the endometrium in 20–38% of women^4^, ^8^. In these cases, ET is likely overestimated, which decreases the specificity of the diagnosis^9^, ^10^.

Common pathologies that affect uterine anatomy, such as fibroids and adenomyosis, have been shown to impede reliable assessment of the endometrium on TVS^8^, ^11^. In addition, an axial position of the uterus results in a suboptimal angle of insonation, which contributes to overestimation of ET measurement. There is some evidence to suggest that secondary imaging modalities, such as transrectal ultrasound (TRS), can more accurately assess the endometrium in women with an axial uterus compared to TVS^11^. Whether alternative sonographic imaging modalities can improve the accuracy of endometrial assessment compared with TVS alone in women presenting with AUB remains to be determined.

The primary objective of this study was to calculate the proportion of TVS assessments of the endometrium in women with AUB that were optimal and, in cases for which TVS assessment was suboptimal, evaluate the efficacy of a multimodal (MM) sonographic approach to imaging the endometrium. This was a part of a larger project that was designed to assess the value of the novel WID‐qEC test for the non‐invasive detection of endometrial cancer^12^.

## METHODS

This was a prospective observational study of a subset of patients who were recruited to participate in the EPI‐SURE study, which evaluated the WID‐qEC epigenetic test for the non‐invasive detection of endometrial cancer and was conducted between June and November 2022 in a tertiary gynecology diagnostic referral center at University College London Hospital, London, UK^12^. All consecutive women aged ≥ 45 years with AUB who underwent TVS assessment of the endometrium were included. AUB was defined as postmenopausal bleeding or irregular vaginal bleeding in women aged ≥ 45 years. This included unscheduled bleeding on hormone replacement therapy. If the endometrium could not be visualized optimally on TVS, women were immediately offered another method of endometrial assessment: transabdominal ultrasound (TAS), TRS or saline contrast sonohysterography (SCSH) (MM approach). All ultrasound examinations were carried out by experienced gynecologists trained in gynecological ultrasound using high‐resolution equipment (Voluson E8; GE Healthcare, Milwaukee, WI, USA) equipped with a 4–9‐MHz transvaginal probe (also utilized for TRS and SCSH) and a 3–8‐MHz transabdominal probe. The same experienced gynecologist performed the initial TVS scan as well as all subsequent second‐, third‐ and fourth‐line imaging tests (as required) during a single clinic visit. All findings and management plans were discussed with Level‐III ultrasound examiners.

The EPI‐SURE study received ethical approval from the UK Health Research Authority (REC 14/LO/1633; IRAS 53431). This study was reported in accordance with the Strengthening the Reporting of Observational Studies in Epidemiology (STROBE) guidelines for observational studies^13^.

### Ultrasound assessment

Ultrasound scans were performed after written informed consent was obtained. Women were examined in the lithotomy position with an empty bladder. In all cases, TVS was performed first to measure ET and to assess endometrial morphology using subjective pattern recognition, as described previously^11^. An ET cut‐off of ≥ 4.5 mm was used to classify women as high risk; these women were then triaged to endometrial biopsy or hysteroscopy, depending on whether focal or global intracavity endometrial abnormality was detected.

The ultrasound operator determined subjectively whether the endometrium was visualized optimally on TVS and whether the angle of insonation was sufficiently close enough to 90°, which was considered optimal for ET measurement. ET was measured in the long‐axis view of the uterus, as close as possible to perpendicular to the endometrial midline echo, and included both endometrial layers at the thickest point. All ultrasound reports and images were stored in our dedicated database (Viewpoint 5.6.25.281; GE Healthcare, Munich, Germany).

If endometrial visualization or ET measurement was considered suboptimal on TVS, another sonographic modality was used, namely TAS or TRS. The choice between TAS and TRS was guided by the cause of suboptimal imaging, patient anatomy and patient preference. TAS was generally used in slim patients with an enlarged uterus and in those with anterior pelvic adhesions fixing the uterus to the abdominal wall. TRS was preferred in overweight patients with a small axial uterus. An axial uterus was defined as a uterine position in which the longitudinal axis of the uterus is parallel to the vaginal axis, resulting in a straightened uterocervical angle. After TVS assessment, the high‐frequency probe was removed, disinfected according to local infection control guidelines and resheathed with a new sterile probe cover and gel before being used to perform TRS following the protocol described previously^11^. A full bladder was not required for TAS because this modality was indicated primarily in patients with an axial uterus, in whom the uterus is often already pulled up towards the anterior abdominal wall, allowing for position correction without the need for a full bladder. If, after TAS or TRS, further assessment of the endometrium was required, SCSH was performed. However, in some cases in which focal lesions were suspected, SCSH was performed as second‐line test instead of TAS or TRS. For SCSH, an 8‐Fr fine soft plastic suction catheter (GBUK Healthcare, Selby, UK) was passed beyond the internal cervical os and 5–10 mL of sterile saline solution was introduced into the uterine cavity, allowing for optimal visualization of the endometrial cavity, as described previously^14^. If all imaging assessments were suboptimal, the patient was advised to undergo endometrial biopsy, hysteroscopy or pelvic magnetic resonance imaging (MRI), based on the presence or absence of intracavitary pathology.

Calculation of the angle of insonation during first‐, second‐ and third‐line sonographic imaging was conducted retrospectively using stored ultrasound images. The ultrasound software's angle‐measurement tool was utilized to measure the angle between the ultrasound beam and the long axis of the endometrium. Two expert ultrasound examiners independently measured the angle and the mean of their measurements was used for analysis.

### Statistical analysis

A previous study by Goldstein and Khafaga^8^ showed that 38% of endometrial assessments on TVS were suboptimal. To test this hypothesis, a *post‐hoc* power calculation was employed, which determined that a sample size of 363 women was required to estimate the expected proportion with a 5% margin of error and 95% confidence^15^.

Demographic and clinical information was collected prospectively at the time of ultrasound assessment using a predefined data collection tool, and from patient healthcare records stored in EPIC (Epic Systems Corp., Verona, WI, USA) and Viewpoint (version 5; Bildverargeritung GmbH, Munich, Germany) software systems. Data collected included age, body mass index (BMI), ethnicity, parity, menopausal status, use of hormone replacement therapy (HRT) and gynecological diagnosis. Patients with a thin endometrium on MM imaging were monitored for a minimum of 12 months following their initial assessment to identify any reattendance or subsequent diagnosis of endometrial cancer.

Data were anonymized and harmonized for analysis, with the primary outcome being optimal (0) or suboptimal (1) ET assessment. Suboptimal endometrial assessment was further divided into overestimation of ET (as a result of an unfavorable uterine position leading to a suboptimal insonation angle) *vs* non‐visualization of the endometrium (due to myometrial abnormality). For independent‐group comparisons (optimal TVS *vs* suboptimal TVS), continuous variables were analyzed using the Mann–Whitney *U*‐test. For paired within‐patient comparisons (suboptimal TVS *vs* optimal MM), continuous variables were analyzed using Wilcoxon's signed‐rank test.

Associations of demographic and clinical factors with suboptimal ET assessment on TVS and MM imaging were investigated using univariable and multivariable logistic regression analyses. Factors showing some association (*P* < 0.20) with the outcome on univariable analysis were carried forward into the multivariable model. Within the multivariable model, a backwards selection procedure was applied, omitting non‐significant variables sequentially until only those with significant independent associations remained. Effect sizes were reported as odds ratios (OR) with 95% CI. For categorical variables, the OR represented the odds of a suboptimal scan in each category relative to the odds in a reference category. Reference categories were defined as follows: ethnicity, white; parity, nulliparous; menopausal status, pre‐/perimenopausal; current HRT use, no; myometrial abnormality, absent. For continuous variables, the OR represented the relative change in the odds of a suboptimal scan for a given increase in that variable.

The specificity of an ET cut‐off of ≥ 4.5 mm to detect endometrial cancer was calculated. The corresponding 95% CI was calculated using the exact binomial method. Participants with missing data were excluded from the specificity analysis.

A *P*‐value < 0.05 was considered statistically significant. GraphPad Prism version 10.2 was used for statistical analysis and generation of graphs.

## RESULTS

A total of 474 women were eligible for the EPI‐SURE study and 400 consented to participate; however, one patient withdrew following consent. Of the remaining 399 women, 11 (2.8%) were excluded because they underwent either TAS (*n* = 8 (2.0%)) or TRS (*n* = 3 (0.8%)) as the first‐line imaging modality. Thus, TVS was used as the first‐line imaging modality to assess the endometrium in 388 women. After exclusion of one case for incomplete data regarding endometrial assessment, we analyzed a total of 387 cases. Demographic and clinical data of the study population are summarized in Table 1.

**Table 1:** Demographic and clinical characteristics of 387 women with abnormal uterine bleeding, overall and according to adequacy of first‐line transvaginal ultrasound (TVS) assessment of endometrium

Characteristic	Overall (*n* = 387)	Optimal TVS (*n* = 245)	Suboptimal TVS (*n* = 142)
Age at recruitment (years)	56 (53–62)	57 (53–63)	56 (52–60)
Parity	2 (1–3)	2 (0–3)	2 (1–3)
0	95 (24.5)	64/95 (67.4)	31/95 (32.6)
1	70 (18.1)	43/70 (61.4)	27/70 (38.6)
2	116 (30.0)	73/116 (62.9)	43/116 (37.1)
3	66 (17.1)	42/66 (63.6)	24/66 (36.4)
≥ 4	40 (10.3)	23/40 (57.5)	17/40 (42.5)
Body mass index (kg/m^2^)	26 (23–31)	26 (22–31)	27 (24–32)
Menopausal status			
Pre‐/perimenopausal	77 (19.9)	42/77 (54.5)	35/77 (45.5)
Postmenopausal	310 (80.1)	203/310 (65.5)	107/310 (34.5)
Current HRT use	134 (34.6)	83/134 (61.9)	51/134 (38.1)
Ethnicity			
White	277 (71.6)	188/277 (67.8)	89/277 (32.1)
Black	52 (13.3)	23/52 (44.2)	29/52 (55.8)
Asian	42 (10.9)	22/42 (52.4)	20/42 (47.6)
Mixed	12 (3.1)	9/12 (75.0)	3/12 (25.0)
Other	4 (1.0)	3/4 (75.0)	1/4 (25.0)
Endometrial thickness*			
< 4.5 mm	288 (74.4)	190/288 (66.0)	98/288 (34.0)
≥ 4.5 mm	75 (19.4)	55/75 (73.3)	20/75 (26.7)
Not measurable	24 (6.2)†	0/24 (0)	24/24 (100)

Data are given as median (interquartile range), *n* (%) or *n*/*N* (%).

* In cases with suboptimal TVS assessment of endometrium, endometrial thickness measurement was obtained from multimodal (MM) imaging approach.

† Due to suboptimal MM imaging (*n* = 22) or patients opting for hysteroscopy over MM approach (*n* = 2). HRT, hormone replacement therapy.

**Figure 1:**
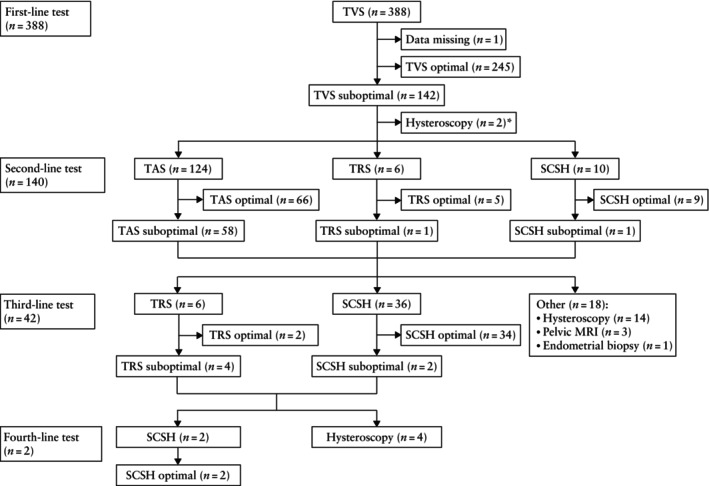
Flowchart summarizing approach to endometrial thickness assessment in 388 women with abnormal uterine bleeding. *Two patients with suboptimal transvaginal ultrasound (TVS) assessment declined additional ultrasound testing and opted for hysteroscopy instead. MRI, magnetic resonance imaging; SCSH, saline contrast sonohysterography; TAS, transabdominal ultrasound; TRS, transrectal ultrasound.

In 245/387 (63.3%) women, the endometrium was visualized optimally on TVS and ET was measured on TVS alone (Figure 1), with a median insonation angle of 82° (interquartile range (IQR), 75–86°). However, ET assessment was suboptimal in the remaining 142/387 (36.7%; 95% CI, 31.8–41.7%) women. The most common standalone reasons for suboptimal TVS were adenomyosis (37/142 (26.1%)) and unfavorable uterine position (37/142 (26.1%)), followed by uterine fibroids (21/142 (14.8%)) and other causes such as the presence of an intrauterine system (IUS) or intrauterine adhesions (6/142 (4.2%)). In 41/142 (28.9%) women, the reason for the suboptimal view on TVS was multifactorial. In the subgroup of 53 women with an unfavorable uterine position, the median insonation angle was 33° (IQR, 15–52°), which was significantly lower compared to that in women with optimal TVS imaging (*P* < 0.0001).

Of the 142 cases for whom endometrial assessment on TVS was considered suboptimal, two patients declined the recommended second‐line sonographic modality (SCSH) and opted for hysteroscopy instead (Figure 1). In the remaining 140 cases, a MM sonographic approach (TAS, TRS and/or SCSH) was employed to further assess the endometrium, which was optimal in 118/140 (84.3%) cases. In the remaining 22/140 (15.7%; 95% CI, 10.1–22.8%) cases, the MM approach was suboptimal, requiring further investigation: endometrial biopsy, pelvic MRI or hysteroscopy. The MM approach enabled optimal endometrial visualization in 49/53 (92.5%) patients with an unfavorable uterine position. The median insonation angle achieved by the MM approach was 86° (IQR, 78–90°) compared with 33° (IQR, 15–52°) on suboptimal TVS in cases with an unfavorable uterine position (*P* < 0.0001). The MM approach corrected non‐visualization of the endometrium due to myometrial abnormality in 69/87 (79.3%) patients.

Overall, when TVS was suboptimal, an optimal ET measurement was achieved on second‐line imaging (TAS, TRS or SCSH) in 80 patients and on third‐line imaging (TRS or SCSH) in a further 36 patients (Figure 1). The total proportion of women with an optimal ET assessment was 325/387 (84.0%) after second‐line testing and 361/387 (93.3%) after third‐line testing. The success of the MM approach was influenced by the presence of myometrial abnormalities. TRS, TAS and SCSH were successful in assessing ET in women with adenomyosis affecting the endometrial–myometrial junction, while SCSH was most effective in women with fibroids distorting the uterine cavity. TAS was particularly beneficial in patients with an unfavorable uterine position that resulted in a suboptimal insonation angle, as it optimized the angle and provided more accurate measurements of ET in 38/41 (92.7%) patients. Although TAS was less effective in cases with myometrial abnormality, it still provided optimal measurements in 28/41 (68.3%) such cases, supporting its role as a valuable second‐line test.

**Figure 2:**
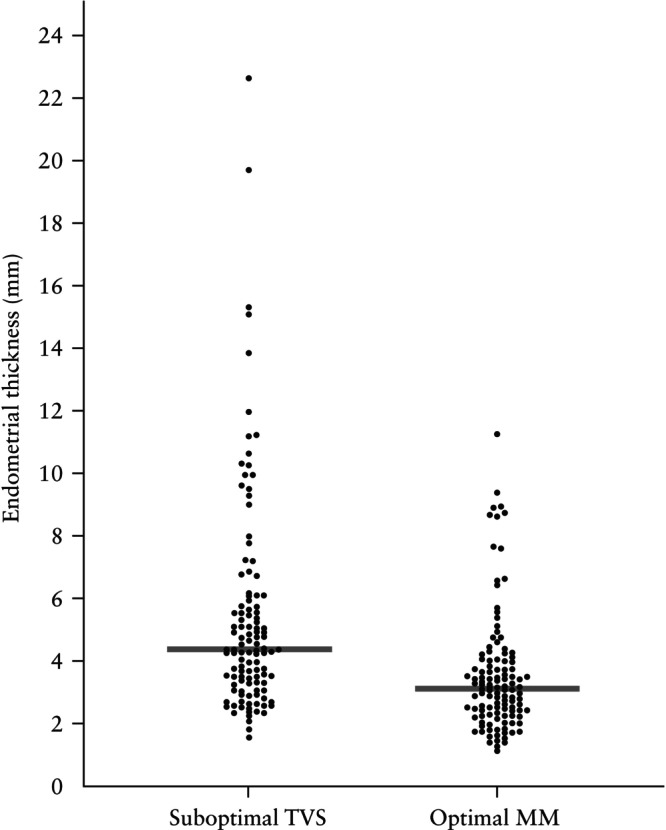
Endometrial thickness measured on suboptimal transvaginal ultrasound (TVS) scans *vs* optimal multimodal (MM) scans in women with abnormal uterine bleeding. Bars indicate medians.

When TVS assessment of the endometrium was considered suboptimal, it was more likely to result in overestimation of ET compared with the MM imaging approach (median difference, −1.3 (95% CI, −1.7 to −0.9) mm; *P* < 0.0001) (Figure 2).

**Figure 3:**
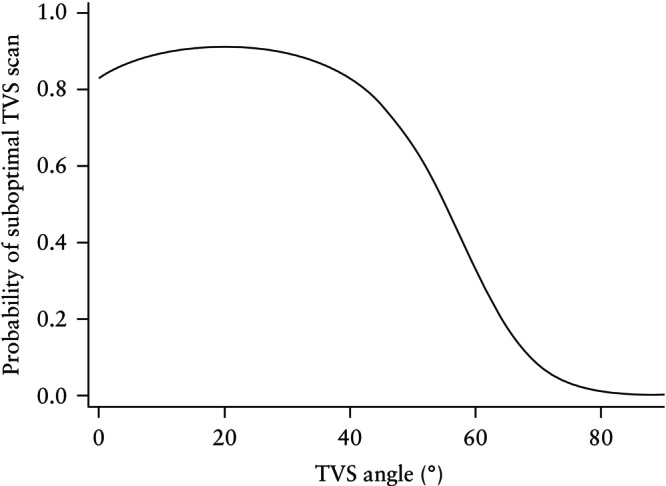
Fitted relationship between the angle of insonation and probability of suboptimal transvaginal ultrasound (TVS) assessment of endometrium in women with abnormal uterine bleeding.

Univariable logistic regression analysis was performed to examine the association between patient characteristics and suboptimal endometrial assessment by TVS (Table 2). When each factor was considered independently, increasing age was linked to decreased odds of suboptimal TVS, with every 5‐year increase in age associated with a 15% reduction in odds. Black and Asian women were more likely to have a suboptimal TVS scan, with over half of Black women (55.8%) and nearly half of Asian women (47.6%) experiencing suboptimal TVS assessment, compared with only 32.1% of White women. The odds of a suboptimal TVS scan in Black women were 2.7 times higher compared with those in White women. Fibroids distorting the uterine cavity were associated strongly with suboptimal TVS; women with fibroids had a 10‐fold increase in the odds of a suboptimal TVS scan compared to those without fibroids. Figure 3 illustrates the relationship between insonation angle and the rate of suboptimal TVS scans. A suboptimal endometrial assessment on TVS was most likely when the angle of insonation was low; as the angle increased beyond 40°, the probability of suboptimal TVS decreased. Postmenopausal status showed a trend towards lower odds of suboptimal TVS, although this did not reach statistical significance. BMI, parity, HRT use and adenomyosis were not associated significantly with suboptimal TVS assessment.

**Table 2:** Univariable and multivariable logistic regression analyses of association between patient characteristics and suboptimal transvaginal ultrasound (TVS) assessment of endometrium in women with abnormal uterine bleeding

Variable	OR (95% CI)	*P*	aOR (95% CI)	*P*
Age at recruitment*	0.85 (0.75–0.98)	0.02	0.86 (0.74–0.99)	0.04
Body mass index†	1.11 (0.97–1.28)	0.13	—	—
Ethnicity		0.004		0.09
White	1 (ref)		1 (ref)	
Black	2.66 (1.46–4.87)		1.87 (0.96–3.64)	
Asian	1.92 (1.00–3.70)		1.93 (0.96–3.85)	
Mixed/other	0.70 (0.22–2.24)		0.71 (0.21–2.41)	
Parity		0.85		—
0	1 (ref)		—	
1	1.30 (0.68–2.47)		—	
2	1.22 (0.69–2.15)		—	
3	1.18 (0.61–2.28)		—	
≥ 4	1.53 (0.71–3.26)		—	
Menopausal status		0.08		—
Pre‐/perimenopausal	1 (ref)		—	
Postmenopausal	0.63 (0.38–1.05)		—	
Current HRT use		0.60		—
No	1 (ref)		—	
Yes	1.09 (0.71–1.69)		—	
Fibroids distorting cavity		< 0.001		< 0.001
No	1 (ref)		1 (ref)	
Yes	10.3 (4.81–22.0)		10.1 (4.64–22.1)	
Adenomyosis affecting EMJ		0.79		—
No	1 (ref)		—	
Yes	1.06 (0.70–1.60)		—	
Insonation angle‡, §		< 0.001		—
Linear term	2.15 (0.97–4.76)		—	
Squared term	0.83 (0.75–0.91)		—	

* Odds ratio (OR) is given per 5‐year increase.

† OR is given per 5‐kg/m^2^ increase.

‡ OR is given per 10° increase.

§ Insonation angle measured in only 63/142 suboptimal TVS scans, so variable was not included in multivariable analysis. aOR, adjusted odds ratio; EMJ, endometrial–myometrial junction; HRT, hormone replacement therapy; ref, reference.

Only the variables that were associated significantly with suboptimal TVS in the univariable analysis were included in the final multivariable model, with the exception of insonation angle, which was excluded because these data were not available for all cases (Table 2). Consistent with the univariable findings, suboptimal TVS was more likely among Black and Asian women compared with White women, but these differences did not reach significance. Women with fibroids were also more likely to experience suboptimal TVS: after adjusting for other variables, the odds of a suboptimal TVS scan were 10 times higher in women with fibroids compared to those without. Conversely, older women were less likely to have a suboptimal TVS scan, with a 14% reduction in odds per 5‐year increase in age.

Univariable logistic regression analysis was also conducted to examine the association between patient characteristics and suboptimal MM imaging of the endometrium (Table 3). Only the presence of cavity‐distorting fibroids was associated significantly with a suboptimal MM scan. There was some evidence to suggest that suboptimal MM imaging was associated with older age, higher BMI, lack of HRT use and absence of adenomyosis; however, these associations did not reach statistical significance. There was no evidence that ethnicity, parity, menopausal status or insonation angle were associated with suboptimal MM assessment of the endometrium. On multivariable analysis, cavity‐distorting fibroids and higher BMI were associated significantly with suboptimal MM imaging. The presence of fibroids increased the odds of a suboptimal MM scan by 4.2 times, while the odds increased by 29% for every 5‐kg/m^2^ increase in BMI.

**Table 3:** Univariable and multivariable logistic regression analyses of association between patient characteristics and suboptimal multimodal (MM) imaging of endometrium in women with abnormal uterine bleeding

Variable	Cases (*n*/*N* (%))	OR (95% CI)	*P*	aOR (95% CI)	*P*
Age at recruitment*	—	1.31 (0.99–1.74)	0.06	1.34 (0.98–1.84)	0.07
Body mass index†	—	1.29 (0.98–1.69)	0.07	1.29 (1.06–1.92)	0.02
Ethnicity			0.48		—
White	16/88 (18)	1 (ref)		—	
Black	4/29 (14)	0.72 (0.22–4.35)		—	
Other	2/23 (9)	0.43 (0.09–2.02)		—	
Parity			0.63		—
0	6/31 (19)	1 (ref)		—	
1	4/27 (15)	0.72 (0.18–2.90)		—	
2	8/42 (19)	0.98 (0.30–3.18)		—	
≥ 3	4/40 (10)	0.46 (0.12–1.81)		—	
Menopausal status			0.79		—
Pre‐/perimenopausal	5/35 (14)	1 (ref)		—	
Postmenopausal	17/105 (16)	1.16 (0.39–3.41)		—	
Current HRT use			0.07		—
No	18/90 (20)	1 (ref)		—	
Yes	4/50 (8)	0.35 (0.11–1.09)		—	
Fibroids distorting cavity			0.004		0.004
No	10/101 (10)	1 (ref)		1 (ref)	
Yes	12/39 (31)	4.04 (1.58–10.4)		4.20 (1.56–11.4)	
Adenomyosis affecting EMJ			0.08		—
No	16/77 (21)	1 (ref)		—	
Yes	6/63 (10)	0.40 (0.15–1.10)		—	
Insonation angle‡, §	—	0.38 (0.07–2.06)	0.26	—	—

* Odds ratio (OR) is given per 5‐year increase.

† OR is given per 5‐kg/m^2^ increase.

‡ OR is given per 10° increase.

§ Insonation angle measured in only 47/140 patients undergoing MM imaging, so variable was not included in multivariable analysis. aOR, adjusted odds ratio; EMJ, endometrial–myometrial junction; HRT, hormone replacement therapy; ref, reference.

We collected follow‐up data on 98 patients with a thin endometrium on the initial assessment. Only one patient was diagnosed with endometrial cancer. She presented with a large hematometra, and although the endometrium appeared thin, there was a strong suspicion of endometrial cancer, which was confirmed by urgent hysteroscopy. Seventy patients visited various hospital departments > 12 months after their assessment in our clinic, none of whom was diagnosed with endometrial cancer. The remaining 27 patients did not attend for any follow‐up visit, gynecological or otherwise. On the basis of the *a‐priori* defined ET cut‐off of ≥ 4.5 mm, and using histology and patients being cancer‐free at > 12‐month follow‐up as reference standards, the specificity of the MM sonographic approach in detecting endometrial cancer was 85.8% (95% CI, 78.0–91.7%). A number needed to treat of 4.8 (95% CI, 3.4–8.2) indicated that approximately five women required assessment with the MM approach to achieve one additional optimal evaluation of ET, particularly when the insonation angle was suboptimal. Given that the MM approach avoided additional invasive procedures to assess the endometrium in 118/387 (30.5%) cases, we conclude that the false‐negative rate was low, and that the MM approach has both high sensitivity and high specificity for the diagnosis of endometrial cancer.

## DISCUSSION

In this study of ET measurement in women with AUB, TVS provided a suboptimal view of the uterine cavity in more than one‐third of cases. In patients with a suboptimal TVS scan, the assessment of endometrial morphology was impaired, often leading to a falsely inflated ET measurement. In such cases, a secondary imaging modality (TAS, TRS or SCSH) can facilitate higher‐quality endometrial imaging and more accurate measurement of ET.

There were two main reasons for suboptimal ET assessment on TVS. First, an unfavorable uterine position often prevented examiners from achieving the optimal angle of insonation to assess and measure the endometrium. Second, uterine abnormalities, most commonly adenomyosis and fibroids, impaired visualization of the endometrium by affecting both uterine position and ultrasound image quality.

**Figure 4:**
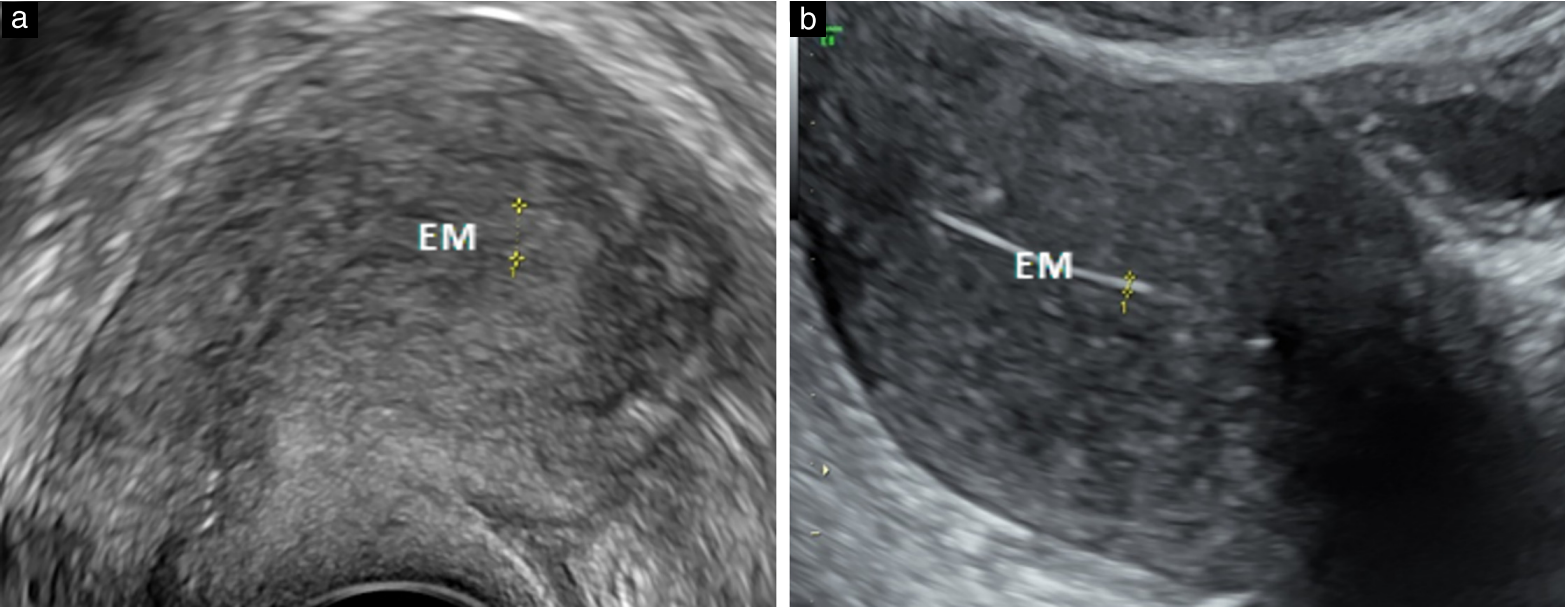
Visualization of endometrium (EM) in the presence of adenomyosis on transvaginal ultrasound (a) and on transabdominal ultrasound (multimodal approach) (b). Calipers indicate EM thickness.

**Figure 5:**
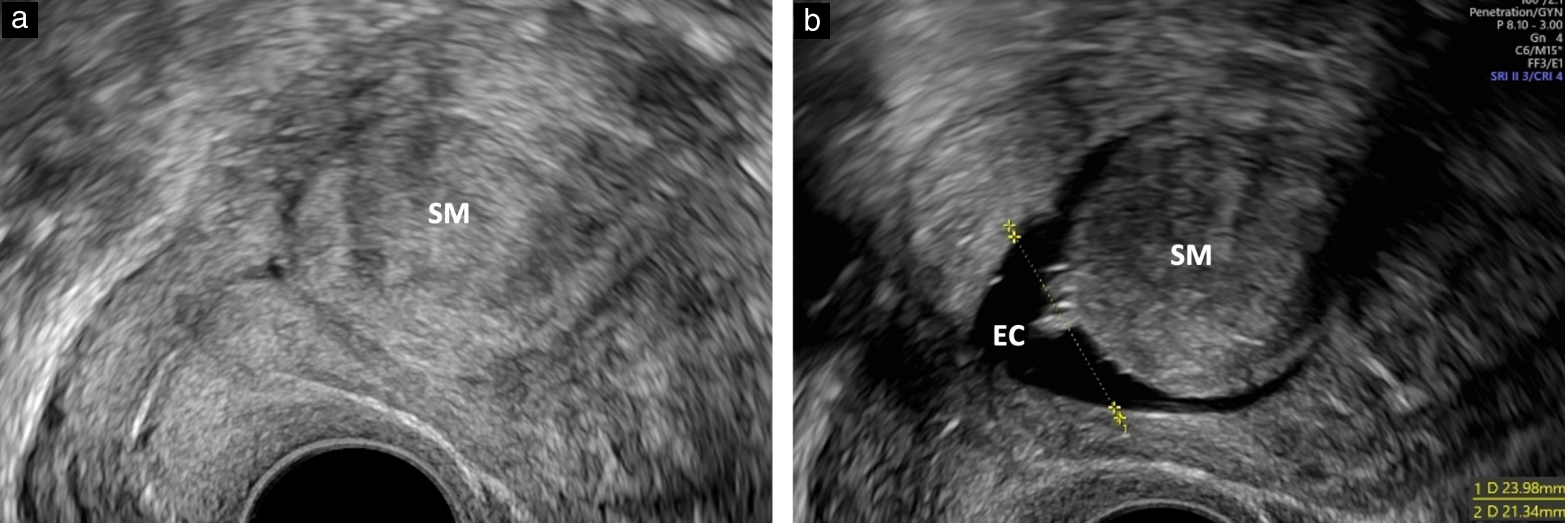
Visualization of endometrium in presence of submucous fibroid (SM) on transvaginal ultrasound (a) and on saline contrast sonohysterography (multimodal approach) (b). Calipers indicate measurement technique. EC, endometrial cavity.

Consistent with our findings, previous studies have shown that an axial uterus^11^, uterine fibroids, adenomyosis and higher BMI contribute to suboptimal visualization of the endometrium on TVS^8^, ^11^, ^16^, ^17^. Adenomyosis and fibroids can distort the endometrial–myometrial junction, impeding accurate measurement (Figures 4 and 5). In our clinical practice, we have observed a strong association between adenomyosis and suboptimal TVS imaging. On univariable analysis, there was a trend toward improved MM imaging following suboptimal TVS in women with adenomyosis, although the small sample size may explain the lack of statistical significance for this result. On multivariable analysis, higher BMI was associated with suboptimal MM imaging. These factors should be considered when triaging patients with AUB based solely on ET.

**Figure 6:**
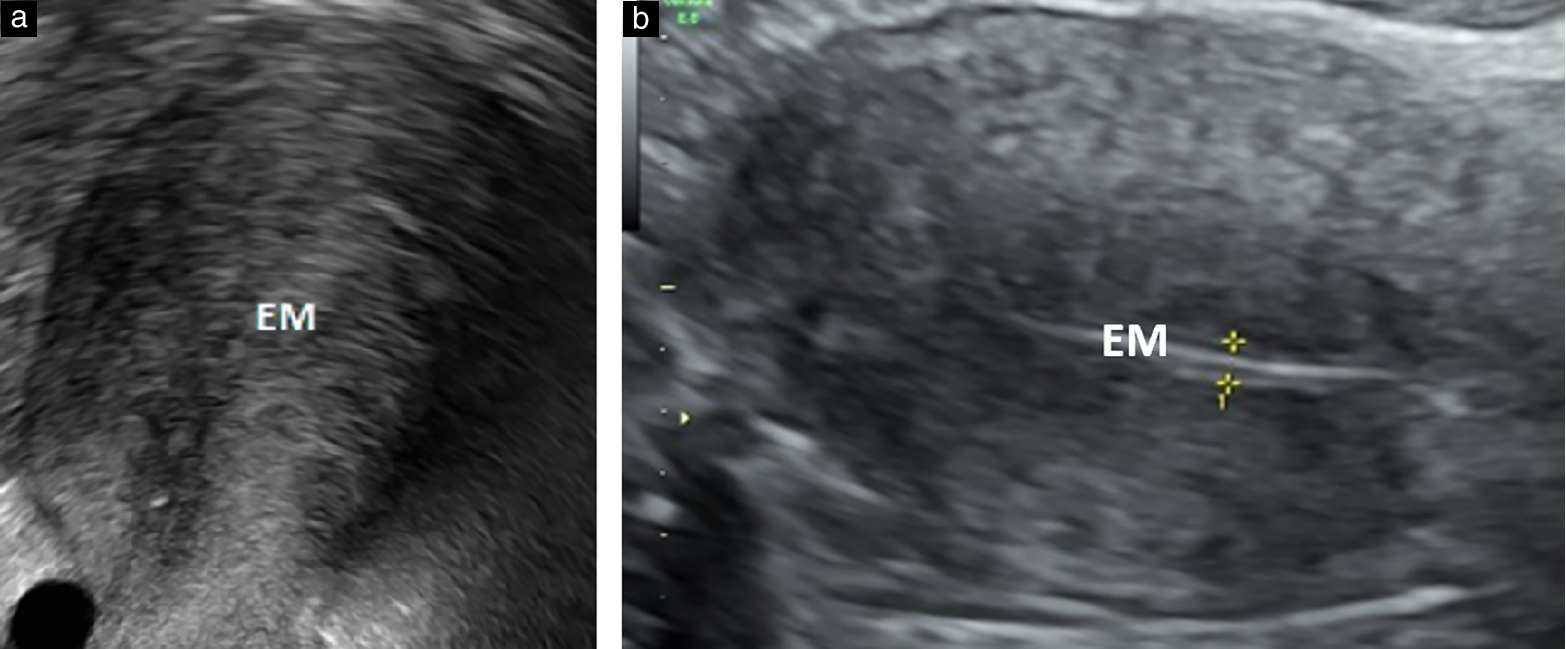
Visualization of endometrium (EM) in uterus with unfavorable position on transvaginal ultrasound (a) and on transabdominal ultrasound (multimodal approach) (b). Calipers indicate EM thickness.

Because of the physical properties of ultrasound, the assessment of ET is more accurate the closer the angle of insonation is to 90°. In an axial uterus, the ultrasound beam is often parallel to the long axis of the endometrium, resulting in an angle of insonation closer to 0° (Figure 6). Divergence of the ultrasound beam combined with the need to examine the endometrium using mainly lateral resolution may result in suboptimal imaging of the endometrium and overestimation of ET.

In the case of suboptimal visualization of the endometrium on TVS, the International Endometrial Tumor Analysis group recommends SCSH as a second‐line test^18^. Although SCSH is effective for excluding intracavitary pathology, such as endometrial polyps, it may not be available in all diagnostic units, may be too painful in women with severe vulvovaginal atrophy and offers little benefit when the cause of suboptimal ET assessment is an unfavorable insonation angle. In these cases, TRS may be more readily available, more acceptable and less painful as a second‐line test compared with SCSH. This is in agreement with a previous study which showed that TRS is associated with a higher proportion of satisfactory endometrial assessments in patients with an axial uterus^11^. In that study, the prevalence of axial uterus in postmenopausal women was 6.6% and TRS was deemed acceptable by most participants, with 64.1% consenting to this imaging modality. Our data demonstrate that an optimal endometrial assessment can be achieved in most women with adenomyosis using TAS or TRS, whereas SCSH is particularly useful for women with fibroids distorting the uterine cavity.

Our study shows that a flexible attitude to imaging modality selection should be exercised to achieve an optimal insonation angle and accurate ET measurement in women presenting with AUB. We found a significant decrease in the mean recorded ET measurement using a MM approach. This had a significant positive effect on the specificity of ultrasound examination for detecting endometrial cancer, which was 85.8% (95% CI, 78.0–91.7%) in our study, without any loss of sensitivity. In comparison, previous studies, which used TVS alone, reported specificity values for endometrial cancer ranging from 35.4% for an ET cut‐off of 3 mm to 52.7% for a cut‐off of ≥ 4 mm and 62.4% for a cut‐off of ≥ 5 mm^3^, ^5^, ^19^. Compared with TVS, the MM sonographic approach is more likely to achieve an optimal angle of insonation, resulting in better visualization of the uterine cavity and lower ET measurements. Since a thinner endometrium is associated with a reduced likelihood of endometrial cancer, the MM approach may improve diagnostic accuracy in detecting malignancy.

High specificity of ultrasound diagnosis is important because it minimizes the use of unnecessary, expensive and/or invasive investigations, such as endometrial biopsy, hysteroscopy and MRI. Recent work highlighted the substantial financial cost of endometrial assessment in women with AUB when the diagnostic pathway includes hysteroscopy, suggesting that alternative, more cost‐effective diagnostic approaches should be investigated^20^. By using a MM approach instead of TVS alone, we were better able to visualize the endometrium and reassure more women during a single clinical visit that there was no sonographic evidence of gynecological malignancy, thereby potentially reducing the psychological distress associated with diagnostic uncertainty, additional testing and follow‐up visits. The MM approach may slightly extend consultation time; however, this could be offset by a reduced need for additional intervention.

Although our study demonstrated promising results, we acknowledge several limitations. The sample size was limited for certain subgroups, such as women with adenomyosis or fibroids who had suboptimal MM imaging, which may affect the strength of our analyses. As the study was conducted at a single center, the findings may not be generalizable to other settings or populations. Additionally, the availability and acceptability of second‐line imaging modalities, such as SCSH and TRS, may vary across clinical environments. Furthermore, we did not stratify by patient‐specific factors that may have influenced endometrial imaging assessment and diagnostic accuracy, such as HRT status and use of hormone‐releasing IUS. Future studies should consider stratifying patients by HRT and IUS use to better understand their impact on endometrial assessment. Moreover, while scan duration was not formally recorded in this study, we recognize that examination time may influence image quality and diagnostic accuracy. Future prospective research should consider incorporating time metrics to better understand their impact on endometrial assessment and to support the standardization of imaging protocols.

We also acknowledge that the study design involves an element of subjective assessment and operator‐dependent decision‐making. While this introduces variability, we believe it reflects real‐world clinical practice where experienced clinicians must make tailored decisions based on factors including uterine position, body habitus and patient comfort. All assessments were conducted by experienced gynecologists using standardized criteria for satisfactory visualization. While subjective, this process mirrors the clinical reasoning underpinning the diagnostic process, aiming to reduce unnecessary invasive procedures and improve patient care. Though the study was conducted prospectively, insonation angle measurements were performed retrospectively by two operators to ensure consistency. We acknowledge that prospective, real‐time evaluation of insonation angle using a standardized and auditable technique would further enhance methodological strength and should be considered in future studies.

In conclusion, the MM sonographic approach is an effective way to measure ET in women with AUB for whom TVS assessment is suboptimal. The MM approach is associated with a higher proportion of optimal ET measurements and may therefore reduce the need for further investigations compared with sole use of TVS. Larger multicenter studies are needed to confirm the validity of our findings, including inter‐ and intraobserver variability and confirmation of serous cancer detection, and to support the recalibration of ET cut‐offs for diagnostic guidelines. Overall, clinicians should be aware of the limitations of TVS in accurately assessing ET and should broaden their skillset to facilitate a more versatile approach to visualizing the endometrium.

## Data Availability

The data that support the findings of this study are available from the corresponding author upon reasonable request.

## References

[1] Jones ER , O'Flynn H , Njoku K , Crosbie EJ . Detecting endometrial cancer. Obstet Gynaecol. 2021;23:103‐112.

[2] Sundar S , Balega J , Crosbie E , et al. BGCS uterine cancer guidelines: recommendations for practice. Eur J Obstet Gynecol Reprod Biol. 2017;213:71‐97. 28437632 10.1016/j.ejogrb.2017.04.015

[3] Long B , Clarke MA , Morillo ADM , Wentzensen N , Bakkum‐Gamez JN . Ultrasound detection of endometrial cancer in women with postmenopausal bleeding: systematic review and meta‐analysis. Gynecol Oncol. 2020;157:624‐633. 32008795 10.1016/j.ygyno.2020.01.032

[4] Wong AS , Lao TT , Cheung CW , et al. Reappraisal of endometrial thickness for the detection of endometrial cancer in postmenopausal bleeding: a retrospective cohort study. BJOG. 2016;123:439‐446. 25800522 10.1111/1471-0528.13342

[5] Timmermans A , Opmeer BC , Khan KS , et al. Endometrial thickness measurement for detecting endometrial cancer in women with postmenopausal bleeding: a systematic review and meta‐analysis. Obstet Gynecol. 2010;116:160‐167. 20567183 10.1097/AOG.0b013e3181e3e7e8

[6] Dueholm M , Marinovskij E , Hansen ES , Møller C , Ørtoft G . Diagnostic methods for fast‐track identification of endometrial cancer in women with postmenopausal bleeding and endometrial thickness greater than 5 mm. Menopause. 2015;22:616‐626. 25535964 10.1097/GME.0000000000000358

[7] Wong M , Thanatsis N , Amin T , Bean E , Madhvani K , Jurkovic D . Ultrasound diagnosis of endometrial cancer by subjective pattern recognition in women with postmenopausal bleeding: prospective inter‐rater agreement and reliability study. Ultrasound Obstet Gynecol. 2021;57:471‐477. 32621381 10.1002/uog.22141

[8] Goldstein SR , Khafaga A . Ability to successfully image endometrium on transvaginal ultrasound in asymptomatic postmenopausal women. Ultrasound Obstet Gynecol. 2021;58:625‐629. 33998081 10.1002/uog.23667

[9] Doll KM , Pike M , Alson J , et al. Endometrial thickness as diagnostic triage for endometrial cancer among Black individuals. JAMA Oncol. 2024;10:1068‐1076. 38935372 10.1001/jamaoncol.2024.1891 PMC11211989

[10] Ken‐Amoah S , Redl E , Domson BKS , et al. Performance of the WID‐qEC test to detect uterine cancers in black women with abnormal uterine bleeding: a prospective observational cohort study in Ghana. Int J Cancer. 2025;156:1055‐1064. 39655721 10.1002/ijc.35260 PMC11701417

[11] Wong M , Amin T , Thanatsis N , Foo X , Jurkovic D . Efficacy of transrectal ultrasound in assessing endometrium of postmenopausal women with axial uterus. Ultrasound Obstet Gynecol. 2022;60:414‐419. 34919769 10.1002/uog.24835

[12] Evans I , Reisel D , Jones A , et al. Performance of the WID‐qEC test versus sonography to detect uterine cancers in women with abnormal uterine bleeding (EPI‐SURE): a prospective, consecutive observational cohort study in the UK. Lancet Oncol. 2023;24:1375‐1386. 37944542 10.1016/S1470-2045(23)00466-7

[13] Vandenbroucke JP , von Elm E , Altman DG , et al. Strengthening the Reporting of Observational Studies in Epidemiology (STROBE): explanation and elaboration. PLoS Med. 2007;4:e297. 17941715 10.1371/journal.pmed.0040297 PMC2020496

[14] Salim R , Lee C , Davies A , Jolaoso B , Ofuasia E , Jurkovic D . A comparative study of three‐dimensional saline infusion sonohysterography and diagnostic hysteroscopy for the classification of submucous fibroids. Hum Reprod. 2005;20:253‐257. 15498782 10.1093/humrep/deh557

[15] Sample Size Calculator for Estimating a Single Proportion . Statulator. https://statulator.com/SampleSize/ss1P.html. Accessed September 1, 2025.

[16] Dietterich C , Klausner K , Check JH . A method to improve false interpretation of the endometrial echo pattern as homogeneous hyperechogenic. Fertil Steril. 2010;94:738‐739. 20097338 10.1016/j.fertnstert.2009.12.025

[17] Skaznik‐Wikiel ME , Jelovsek JE , Andrews B , Bradley LD . Accuracy of endometrial thickness in detecting benign endometrial pathology in postmenopausal women. Menopause. 2010;17:104‐108. 19587611 10.1097/gme.0b013e3181ae20de

[18] Leone FP , Timmerman D , Bourne T , et al. Terms, definitions and measurements to describe the sonographic features of the endometrium and intrauterine lesions: a consensus opinion from the International Endometrial Tumor Analysis (IETA) group. Ultrasound Obstet Gynecol. 2010;35:103‐112. 20014360 10.1002/uog.7487

[19] Emons G , Steiner E , Vordermark D , et al. Endometrial Cancer. Guideline of the DGGG, DKG and DKH (S3‐Level, AWMF Registry Number 032/034‐OL, September 2022). Part 1 with Recommendations on the Epidemiology, Screening, Diagnosis and Hereditary Factors of Endometrial Cancer, Geriatric Assessment and Supply Structures. Geburtshilfe Frauenheilkd. 2023;83:919‐962. 37588260 10.1055/a-2066-2051 PMC10427205

[20] Warring SK , Borah B , Moriarty J , et al. The cost of diagnosing endometrial cancer: quantifying the healthcare cost of an abnormal uterine bleeding workup. Gynecol Oncol. 2022;164:93‐97. 34756471 10.1016/j.ygyno.2021.10.079 PMC8724459

